# Simulation Optimization and Application of Shearer Strapdown Inertial Navigation System Modulation Scheme

**DOI:** 10.3390/s23094290

**Published:** 2023-04-26

**Authors:** Gang Wu, Xinqiu Fang, Yang Song, Ningning Chen, Minfu Liang, Jiaxuan Li, Fukang Qiao

**Affiliations:** 1 School of Mines, China University of Mining and Technology, Xuzhou 221116, China; tbh236@cumt.edu.cn (G.W.); song.yang@cumt.edu.cn (Y.S.); ts15020009a3tm@cumt.edu.cn (N.C.); liangmf2014@cumt.edu.cn (M.L.); ts21020122p21@cumt.edu.cn (J.L.); ts22020082a31@cumt.edu.cn (F.Q.); 2 Research Center of Intelligent Mining, China University of Mining and Technology, Xuzhou 221116, China

**Keywords:** shearer, strapdown inertial navigation system (SINS), attitude perception, error compensation, uniaxial rotation modulation

## Abstract

The operating attitude of a shearer based on a three-dimensional (3D) space scale is the necessary basic information for realizing intelligent mining. Aiming to address the problem of the insufficient perception accuracy of shearers, in this paper, the rotation model of the actual turning mechanism of the strapdown inertial navigation system (SINS) of shearers is established, and the error propagation characteristics of different single-axis rotation modulation schemes are revealed. Through theory and simulation, the optimal rotation modulation scheme is determined to be the improved four-position turn–stop modulation with a rotation of <360°. The experiment shows that the 24 h positioning error of this scheme is 3.7 nmile, and the heading angle changes by 0.06°, which proves that this scheme can effectively improve the attitude perception accuracy of the inertial navigation system (INS). The field application of the shearer operating attitude perception based on this scheme shows that the positioning error after error compensation is 17% of that before compensation, and the heading angle error is 75% of that before compensation, which verifies that this scheme can significantly improve the accuracy of shearer operating attitude perception in field applications. This scheme can achieve higher precision perception accuracy based on SINS and has broad application prospects in the field of high-precision pose perception of coal mining machines, roadheaders, and other equipment.

## 1. Introduction

The operating posture of a shearer, including the heading angle, pitch angle, roll angle, and 3D acceleration of the body and cutting part, is based on the three degrees of freedom dynamic information on the 3D space scale, which can determine the position of the shearer and predict the 3D extension direction of the working face in the coal-bearing geological space. The operating posture is also the basic information for realizing the shearer’s memory cutting, automatic height adjustment, intelligent control, intelligent perception, remote visual monitoring [1,2], etc. As a difficult problem of intelligent mining equipment, accurate perception of the shearer’s running attitude can not only provide a way to detect and predict the production status of the intelligent chemical working face but also provide data feedback for the autonomous intelligent control of the shearer. Therefore, it is of great significance for the construction of an intelligent working face to realize the high-precision position and attitude state perception of the shearer based on rotary SINS.

Since the 1990s, traditional coal mining technology powers such as the United States, Germany, and Australia have begun to explore and research intelligent coal mining technology and equipment, and they initially realized remote sensing of coal mining equipment. In 2001, the Commonwealth Science and Industries Research Organization (CSIRO) designed and developed the Longwall Automation Steering Committee (LASC) system with the technical feature of the accurate positioning of coal mining equipment. This system uses the military-level high-precision fiber-optic gyroscope and an exclusively developed navigation positioning algorithm to achieve the accurate positioning of coal mining machines and automatic straightening of the working face scraper [3,4,5], and it has been successfully applied to the Beltana Coal Mine in Australia. In 2008, the LASC system was upgraded and, combined with detailed geological data and a 3D model of the mining space, it achieved the goals of monitoring the position of production equipment in the 3D space, memory cutting of shearers, 3D visualization of the mining space, etc. [6]. At present, LASC has been applied in more than 70% of longwall coal mining faces in Australia. CSIRO released LASC 2.0 in 2017, and signed license agreements with third-party OEM coal machinery equipment manufacturers, such as Bucyrus, JOY, Eickhoff, Tiandi Technology, etc. CSIRO and coal machinery manufacturers jointly promoted LASC, realizing rapid commercialization [7,8,9,10].

As the main sensor in the LASC system, the gyroscope was developed over a period of more than 170 years, and its accuracy means that it is difficult to achieve a big breakthrough [11,12]. As an error self-compensation method of INS, the essence of rotary modulation is to generate periodic attitude matrix changes through an external turning mechanism without improving the hardware level of the inertial sensor, thereby achieving self-compensation of the deterministic error of the inertial sensor [13,14] and improving the system accuracy for the long voyage. Scholars have been researching rotary navigation systems since the 1980s. Because of its simple structure and good error compensation effect, the rotary fiber-optic INS has great potential to replace the existing laser gyroscope, electrostatic gyroscope, and other types of gyroscope [15]. Moreover, most of the existing high-precision SINS products also use rotary modulation to improve navigation accuracy. Thus far, many scholars have conducted much useful research work on initial alignment [16,17,18], error modulation [19], rotation modulation schemes [20,21], solution algorithms [22,23], etc., and achieved rich research results. However, as one of the important application scenarios of SINS, there is little research on the rotation error modulation and field applicability of shearers. In addition, the shearer is subjected to the combined action of large loads and strong vibrations during the reciprocating coal-cutting process. At the same time, since the inertial sensors in the SINS used in coal mines are directly fixed to the shearer, they need to directly withstand the various impacts, vibrations, and noise disturbances caused by coal mining operations [24,25]. That is, the working conditions are usually not ideal. In addition, the shearer will start and stop multiple times within a day, and as the working time increases, the gyroscope will accumulate significant errors. There will be significant static errors; therefore, the SINS has put forward higher requirements for the performance of inertial sensitive devices.

This paper focuses on the insufficient accuracy of the current coal mine SINS and the lack of a basis for selecting rotation modulation schemes. Based on the analysis of the inertial measurement unit (IMU) error modulation mechanism, this paper optimizes the optimal rotational modulation scheme by simulating and analyzing the long-term positioning error of each scheme. Based on this, laboratory experiments and industrial applications were conducted. Compared with the SINS without error modulation, the optimal modulation scheme of the shearer SINS selected in this paper can effectively reduce the positioning error of the underground coal mining machine and tunneling machine. This scheme has great engineering application value and application prospects in intelligent mining processes.

## 2. Basic Principle of Rotation Modulation Technology

**Figure 1:**
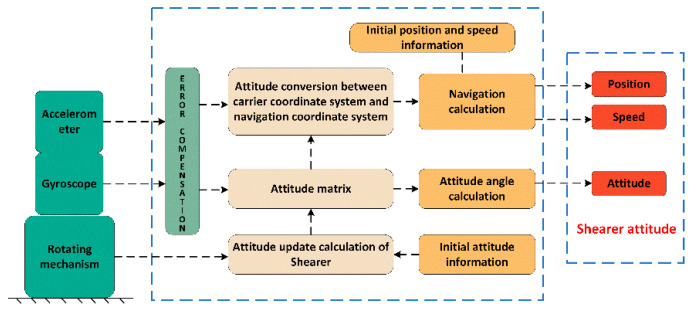
Principle of shearer rotary INS.

The rotary INS fixedly connects the IMU with the shearer through the turning mechanism. The IMU can rotate periodically with the turning mechanism. The navigation solution also uses the SINS algorithm. In this way, the attitude information calculated by the navigation is only the attitude of the IMU. At this time, the attitude information of the shearer can be obtained by taking into account the rotation angle of the IMU relative to the shearer [26]. The basic principle is shown in Figure 1.

SINS mainly uses a carrier coordinate system (*b* system), navigation coordinate system (*n* system), rotation coordinate system (*s* system), and inertial coordinate system (*i* system) in the attitude calculation process. When the IMU remains stationary at the initial time, the *s* system, *b* system, and *n* system remain coincident, the origin is located in the center of the IMU, and the coordinate axis refers to the three orthogonal axes of the IMU.

IMU starts at angular speed *ω* and rotates around the celestial axis, and the shearer remains stationary during rotation, so at time *t*, the included angle between the *x*-axes of the two coordinate systems is *β* = *ωt*. At this time, the direction cosine array of the *b* system relative to the *s* system is as follows:

(1) $$C_{b}^{s}=\left[\begin{array}{ccc} \cos\beta & \sin\beta & 0\\ -\sin\beta & \cos\beta & 0\\ 0 & 0 & 1 \end{array}\right]=\left[\begin{array}{ccc} \cos\omega t & \sin\omega t & 0\\ -\sin\omega t & \cos\omega t & 0\\ 0 & 0 & 1 \end{array}\right]={\left(C_{s}^{b}\right)}^{T},$$

Assuming that the ideal angular velocity and specific force of system *b* relative to system *i* are $\omega_{ib}^{b}$ and $f^{b}$, respectively, the ideal output of IMU gyroscope and accelerometer is as follows:

(2) $$\left\{\begin{array}{l} \omega_{is}^{s}=C_{b}^{s}\omega_{ib}^{b}+\omega_{bs}^{s}\\ f^{s}=C_{b}^{s}f^{b}+f_{bs} \end{array}\right.,$$

where $\omega_{bs}^{s}={\left[0\;0\;\omega \right]}^{T}$ and $f_{bs}={\left[0\;0\;0\right]}^{T}$.

Under the excitation of angular velocity $\omega_{is}^{s}$ and specific force $f^{s}$, the output of the gyroscope and accelerometer is as follows:

(3) $$\left\{\begin{array}{l} \omega_{is}^{s}=\left(I+\delta K_{g}\right)\left(I+\delta E_{g}\right)\omega_{is}^{s}+\varepsilon_{n}+\varepsilon_{r}\\ f^{s}=\left(I+\delta K_{a}\right)\left(I+\delta E_{a}\right)f^{s}+\nabla_{n}+\nabla_{r} \end{array}\right.,$$

where $\omega_{is}^{s}$ is the gyro output value, deg/s; $f^{s}$ is the accelerometer output value, g; $\delta K_{g}$ is the gyro scale factor error, ppm; $\delta K_{a}$ is the accelerometer scale factor error, mA/g; $\delta E_{g}$ is the gyro installation error, deg; $\delta E_{a}$ is the accelerometer installation error, deg; $\omega_{is}^{s}$ is the ideal gyro input value, deg/s; $f^{s}$ is the accelerometer ideal input value, g; $\varepsilon_{n}$ is the gyro random drift error, deg; and $\nabla_{n}$ is the accelerometer random drift error, g.

After expanding Equation (3), subtract the ideal output value of the inertial sensor and omit the second order small quantity. Then, the output error can be obtained as follows:

(4) $$\left\{\begin{array}{l} \delta \omega_{is}^{n}=C_{s}^{n}\left[\left(\delta K_{g}+\delta E_{g}\right)\left(C_{b}^{s}\omega_{ib}^{b}+\omega_{bs}^{s}\right)+\varepsilon_{n}+\varepsilon_{r}\right]\\ \delta f^{n}=C_{s}^{n}\left[C_{b}^{s}\left(\delta K_{a}+\delta E_{a}\right)f^{s}+\nabla_{n}+\nabla_{r}\right] \end{array}\right.,$$

where $C_{s}^{n}=C_{b}^{n}C_{s}^{b}$ and $C_{s}^{b}={\left(C_{b}^{s}\right)}^{T}$.

According to Formula (4), after the IMU rotates 360° relative to the shearer, the end value of the east and north gyro drift is 0; that is, the constant error of the inertial sensor perpendicular to the rotation axis is offset, which will not affect the accuracy of the navigation system.

## 3. Analysis of Rotary Modulation Scheme and Mechanism

### 3.1. Single-Axis Continuous Rotation Modulation

#### 3.1.1. Single-Axis Continuous Rotation Modulation Scheme

**Figure 2:**
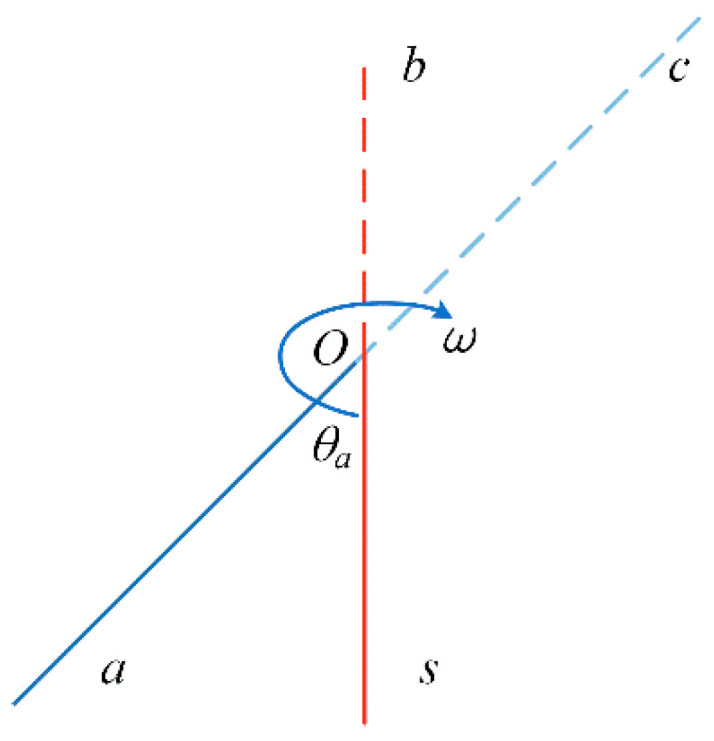
Single-axis continuous rotation modulation scheme.

The *s* system, *b* system, and *n* system coincide at the initial time, and the single-axis continuous rotation modulation scheme is shown in Figure 2.

1. The turning mechanism starts from point *s* at zero time, accelerates to point a through time *t_a_* with angular acceleration α, and the rotation angle during acceleration is *θ_a_*;
2. After the angular velocity reaches the set value *ω*, it will rotate continuously at a constant speed.

#### 3.1.2. Modulation Mechanism Analysis of Single-Axis Continuous Rotation

The continuous rotation time of a single axis is $t=\frac{2\pi n}{\omega }+t_{a}$, and the attitude angle error caused by gyro bias drift is given as follows:

(5) $$\Psi =\left[\begin{array}{c} \varepsilon_{x}^{s}t_{a}-\varepsilon_{y}^{s}\frac{\alpha t_{a}^{3}}{6}\\ \varepsilon_{x}^{s}\frac{\alpha t_{a}^{3}}{6}-\varepsilon_{y}^{s}t_{a}\\ \varepsilon_{z}^{s}t \end{array}\right],$$

where $\varepsilon_{x}^{s}$ is the gyro *x*-axis drift, deg/h; $\varepsilon_{y}^{s}$ is the gyro *y*-axis drift, deg/h; and $\varepsilon_{z}^{s}$ is the gyro *z*-axis drift, deg/h.

The attitude angle error caused by the gyro scale factor error is as follows:

(6) $$\Psi =\left[\begin{array}{c} \frac{\left(K_{gx}-K_{gy}\right)\omega_{ie}\cos\varphi }{6}\alpha t_{a}^{3}\\ K_{gy}\cos\varphi t_{a}+\frac{\left(t-t_{a}\right)\omega_{ie}\cos\varphi }{2}\left(K_{gx}+K_{gy}\right)\\ K_{gz}\left(\omega_{ie}\sin\varphi t_{a}+\frac{1}{2}\alpha t_{a}^{2}\right)+K_{gz}\left(\omega_{ie}\sin\varphi +\omega \right)\left(t-t_{a}\right) \end{array}\right],$$

where *K_gx_* is the *x*-axis scale factor error of the gyroscope, ppm; *K_gy_* is the *y*-axis scale factor error of the gyroscope, ppm; *K_gz_* is the *z*-axis scale factor error of the gyroscope, ppm; *ω_ie_* is the earth angular velocity, deg/s; and *φ* is the latitude, deg.

The attitude angle error caused by the gyro installation error is calculated as follows:

(7) $$\begin{array}{l} \Psi_{x}=\omega_{ie}\cos\varphi E_{gxy}t_{a}+\omega_{ie}\sin\varphi E_{gxz}t_{a}-\omega_{ie}\sin\varphi \frac{\alpha t_{a}^{3}}{6}E_{gyz}+E_{gxz}\sin\frac{1}{2}\alpha t_{a}^{2}\\ +E_{gyz}\left(\cos\frac{1}{2}\alpha t_{a}^{2}-1\right)-\frac{\omega_{ie}\cos\varphi \sin2\theta_{a}}{4\omega }\left(-E_{gxy}+E_{gyx}\right)\\ +\frac{\left(t-t_{a}\right)\omega_{ie}\cos\varphi }{2}\left(E_{gxy}-E_{gyx}\right)-\frac{\omega_{ie}\sin\varphi }{\omega }\left(E_{gxz}\sin\theta_{a}+E_{gyz}\cos\theta_{a}\right)\\ -E_{gxz}\sin\theta_{a}-E_{gyz}\cos\theta_{a} \end{array},$$

where *E_gxy_*, *E_gxz_*, *E_gyx_*, *E_gyz_*, *E_gzx_*, and *E_gzy_* are the installation error angles of the gyroscope, deg.

### 3.2. Single-Axis Continuous Positive and Negative Rotation Modulation

#### 3.2.1. Single-Axis Continuous Positive and Negative Rotation Modulation Scheme

**Figure 3:**
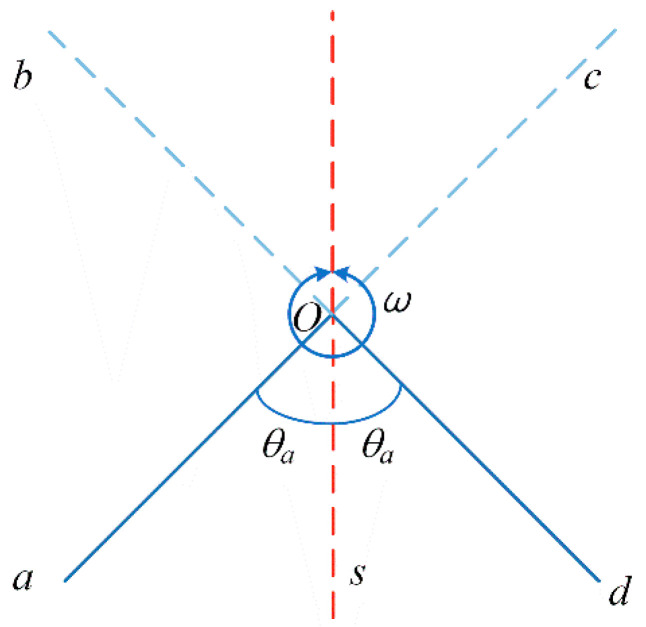
Single-axis continuous positive and negative rotation modulation scheme.

The single-axis continuous positive and negative rotation modulation scheme is shown in Figure 3.

1. The turning mechanism starts from point *s* at zero time and rotates to point *a* with angular acceleration *α* to reach the set angular velocity *ω*. The rotation angle of the process is *θ_a_* and the duration is *t_a_*;
2. Then, rotate to point *d* at a constant speed of angular velocity *ω*. The rotation angle of this process is 2*π* − 2*θ_a_* and the duration is *t_c_* = (2*π* − 2*θ_a_*)/*ω*;
3. Then, decelerate to the starting point *s* with the angular acceleration −*α*. The rotation angle is *θ_a_* and the residence time is *t_s_*;
4. Accelerate to point *d* with acceleration *α* again to reach the set angular velocity −*ω*;
5. Then, rotate to point *a* at a constant speed with angular speed −*ω*;
6. Then, rotate to the starting point *s* at a constant deceleration of angular acceleration *α*, and the dwell time is *t_s_*.

#### 3.2.2. Modulation Mechanism Analysis of Single-Axis Continuous Positive and Negative Rotation

The modulation period of single-axis continuous positive and negative rotation is as follows:

(8) $$T=4t_{a}+2t_{c}+2t_{s},$$

During this period, the attitude angle error caused by gyro bias drift is as follows:

(9) $$\Psi_{r}=\left[\begin{array}{c} 4\varepsilon_{x}^{s}t_{a}-\frac{4\sin\theta_{a}}{\omega }\varepsilon_{x}^{s}+2\varepsilon_{x}^{s}t_{s}\\ 4\varepsilon_{y}^{s}t_{a}-\frac{4\sin\theta_{a}}{\omega }\varepsilon_{y}^{s}+2\varepsilon_{y}^{s}t_{s}\\ \varepsilon_{z}^{s}T \end{array}\right],$$

The attitude angle error caused by the gyro scale factor error is as follows:

(10) $$\Psi_{rKg}=\left[\begin{array}{c} 0\\ \Psi_{ry}\\ K_{gz}\omega_{ie}\sin\varphi T \end{array}\right],$$

where $\Psi_{ry}=2K_{gy}\omega_{ie}\cos\varphi \left(t_{a}+t_{s}\right)+\left(t_{c}+t_{a}\right)\omega_{ie}\cos\varphi \left(K_{gx}+K_{gy}\right)+\omega_{ie}\cos\varphi \left(-K_{gx}+K_{gy}\right)\left(\frac{-\sin2\theta_{a}}{\omega }+2t_{a}\right)$.

The attitude angle error caused by the gyro installation error is as follows:

(11a) $$\begin{array}{l} \Psi_{rEgx}=\left(\omega_{ie}\cos\varphi E_{gxy}+\omega_{ie}\sin\varphi E_{gxz}\right)\left(4t_{a}+2t_{s}\right)\\ \;\;\;+t_{rc}\omega_{ie}\cos\varphi \left(E_{gxy}-E_{gyx}\right)+4E_{gyz}\left(\cos\frac{1}{2}\alpha t_{a}^{2}-1\right) \end{array},$$

(11b) $$\Psi_{rEgy}=4\omega_{ie}\sin\varphi E_{gyz}t_{a}+4E_{gxz}\left(\cos\frac{1}{2}\alpha t_{a}^{2}-1\right)+2\omega_{ie}\sin\varphi E_{gyz}t_{s},$$

(11c) $$\Psi_{rEgz}=E_{gzy}\omega_{ie}\cos\varphi \left(4t_{a}+2t_{s}\right),$$

### 3.3. Four-Position Turn–Stop Modulation with Rotation >360°

#### 3.3.1. Four-Position Turn–Stop Modulation Scheme with Rotation >360°

**Figure 4:**
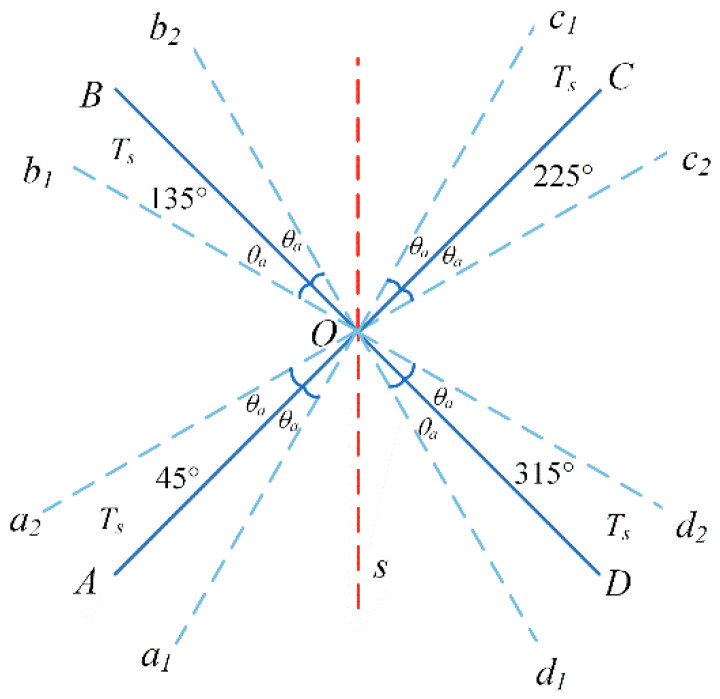
Four-position turn–stop scheme with rotation >360°.

The four-position turn–stop modulation scheme with a rotation of >360° is shown in Figure 4. The residence positions and order of the turning mechanism are as follows: 45°→135°→225°→315°→45°→315°→225°→135°→45°. These positions are completely symmetrical with the acceleration and deceleration processes, and the residence time of each residence position is also the same.

1. The turning mechanism starts from point *A*, with angular acceleration *α* accelerating the rotation to point *a*_2_ to reach the set angular velocity *ω*. The process rotation angle is *θ_a_*;
2. Then, rotate to point *b*_1_ at a constant speed with an angular velocity *ω*, and the rotation angle of the process is 0.5*π* − 2*θ_a_*;
3. Then, decelerate to point *B* with angular acceleration −*α*. The rotation angle is *θ_a_* and the residence time is *t_s_*;
4. Then, rotate to points *C*, *D*, and *A* in the same way, and the residence time at each point is still *t_s_*;
5. Then, reverse the rotation of points *B*, *C*, *D*, and *A* in the same way to form a complete modulation cycle.

#### 3.3.2. Modulation Mechanism Analysis of Four-Position Turn–Stop with Rotation >360°

The modulation period of this scheme is as follows:

(12) $$T=16t_{a}+8t_{c}+8t_{s},$$

where *t_a_* is the acceleration time between two retention points, s; *t_c_* is the uniform rotation time between two retention points, s; and *t_s_* is the residence time at each retention point, s.

The attitude angle error caused by gyro bias drift is as follows:

(13) $$\Psi_{r\varepsilon }=\left[\begin{array}{c} 0\\ 0\\ \varepsilon_{z}^{s}T \end{array}\right],$$

The attitude angle error caused by the gyroscope scale factor error is as follows:

(14) $$\Psi_{rKg}=\left[\begin{array}{c} 0\\ 0.5\omega_{ie}\cos\varphi \left(K_{gx}+K_{gy}\right)T\\ K_{gz}\omega_{ie}\sin\varphi T \end{array}\right],$$

The attitude angle error caused by the gyroscope installation error is as follows:

(15) $$\Psi_{rEg}=\left[\begin{array}{c} \frac{1}{2}\omega_{ie}\cos\varphi \left(E_{gxy}+E_{gyx}\right)T\\ 0\\ K_{gz}\omega_{ie}\sin\varphi T \end{array}\right],$$

In summary, the four-position turn–stop modulation scheme with a rotation of >360° has a very good error modulation effect, which can significantly improve the accuracy of SINS.

### 3.4. Four-Position Turn–Stop Modulation with Rotation <360°

#### 3.4.1. Four-Position Turn–stop Modulation Scheme with Rotation <360°

**Figure 5:**
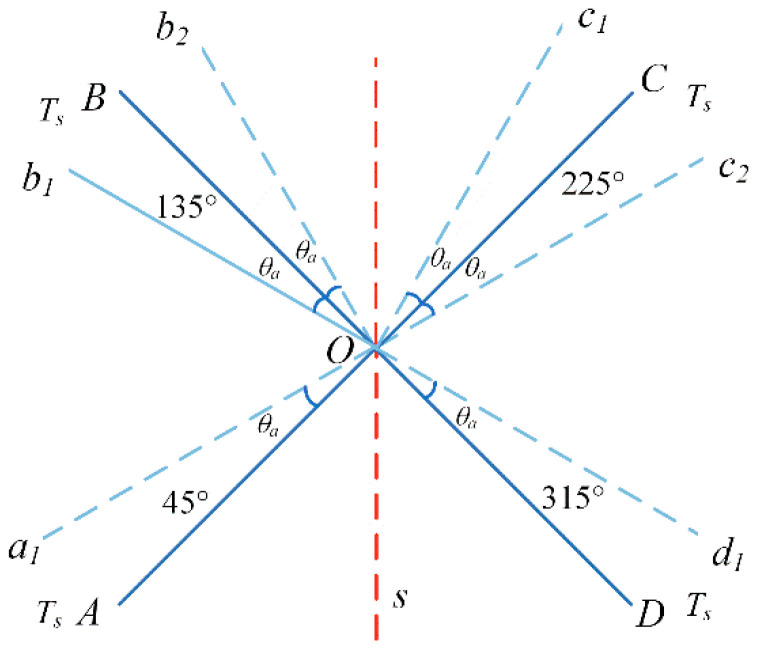
Four-position turn–stop scheme with rotation <360°.

The four-position turn–stop error modulation scheme with rotation <360° is shown in Figure 5. The residence positions and order of the turning mechanism are as follows: 45°→135°→225°→315°→45°. The residence positions are completely symmetrical, but the rotation angle is not completely symmetrical.

1. The turning mechanism starts from point *A*, with angular acceleration *α* accelerating the rotation to point *a*_1_ to reach the set angular velocity *ω*. The process rotation angle is *θ_a_*;
2. Then, rotate to point *c*_1_ at a constant speed, and the rotation angle is *π* − 2*θ_a_* and the rotation time is *t_c_*_1_ = (*π* − 2*θ_a_*)/*ω*;
3. From point *c*_1_, decelerate uniformly to point *C* with angular acceleration −*α*, and the rotation angle during this process is *θ_a_*;
4. Accelerate the rotation from point *C* to point *c*_2_ at the angular acceleration *α* to reach the set angular speed *ω*, and the rotation angle during this process is *θ_a_*;
5. Thereafter, it continuously rotates to point *d*_1_ at a constant speed. The rotation angle in this process is 0.5*π* − 2*θ_a_*, and the rotation time is *t_c_*_2_ = (0.5*π* − 2*θ_a_*)/*ω*. It decelerates uniformly from point *d*_1_ to point *D* at an angular acceleration of −*α*. The rotation angle in this process is *θ_a_*;
6. Then, reverse the rotation of points *B* and *A* in the same way to form a complete modulation cycle, and the residence time of points *A*, *B*, *C*, and *D* is *t_s_*.

#### 3.4.2. Modulation Mechanism Analysis of Four-Position Turn–Stop with Rotation <360°

The modulation period of this scheme is as follows:

(16) $$T=8t_{a}+4t_{s}+2t_{c1}+2t_{c2},$$

The attitude angle error caused by gyro bias drift is as follows:

(17) $$\Psi_{r\varepsilon }=\left[\begin{array}{c} -\frac{\sqrt{2}}{3}\alpha t_{a}^{3}\varepsilon_{x}^{s}-\frac{2\sqrt{2}}{\omega }\cos\left(\theta_{a}\right)\varepsilon_{x}^{s}\\ -\frac{\sqrt{2}}{3}\alpha t_{a}^{3}\varepsilon_{y}^{s}--\frac{2\sqrt{2}}{\omega }\cos\left(\theta_{a}\right)\varepsilon_{y}^{s}\\ \varepsilon_{z}^{s}T \end{array}\right],$$

The attitude angle error caused by the gyroscope scale factor error is as follows:

(18) $$\Psi_{rKg}=\left[\begin{array}{c} -\frac{2}{\omega }\left(K_{gx}-K_{gy}\right)\omega_{ie}\cos\varphi \sin\left(2\theta_{a}\right)\\ \frac{1}{2}\omega_{ie}\cos\varphi \left(K_{gx}+K_{gy}\right)T+\omega_{ie}\cos\varphi \frac{2\alpha t_{a}^{3}}{3}\left(K_{gx}-K_{gy}\right)\\ K_{gz}\omega_{ie}\sin\varphi T \end{array}\right],$$

The attitude angle error caused by the gyroscope installation error is as follows:

(19a) $$\begin{array}{l} \Psi_{rEgX}=\frac{1}{2}\omega_{ie}\cos\varphi \left(E_{gxy}-E_{gyx}\right)T-\left(\frac{1}{\omega }\cos\left(2\theta_{a}\right)+\frac{2}{3}\alpha t_{a}^{3}\right)\\ \omega_{ie}\cos\varphi \left(E_{gxy}+E_{gyx}\right)-\omega_{ie}\sin\varphi E_{gxz}\left(\frac{2\sqrt{2}}{\omega }\cos\left(\theta_{a}\right)\right)-2\sqrt{2}\sin\left(\theta_{a}\right)E_{gyz} \end{array},$$

(19b) $$\Psi_{rEgy}=4\omega_{ie}\sin\varphi E_{gyz}t_{a}+4E_{gxz}\left(\cos\frac{1}{2}\alpha t_{a}^{2}-1\right)+2\omega_{ie}\sin\varphi E_{gyz}t_{s},$$

(19c) $$\Psi_{rEgz}=E_{gzy}\omega_{ie}\cos\varphi \left(4t_{a}+2t_{s}\right),$$

### 3.5. Improved Four-Position Turn–Stop Modulation with Rotation <360°

According to the analysis in Section 3.4, it can be seen that the gyroscope *ε* is not completely compensated in the four-position turn–stop modulation scheme with a rotation of <360°. From Equation (17), the ratio of residual zero-bias drift to total drift is as follows:

(20) $$\begin{array}{ll} Rate & =\frac{\int \varepsilon_{E}^{n}dt}{T\varepsilon_{x}^{s}}=\frac{-\frac{\sqrt{2}}{3}\alpha t_{a}^{3}\varepsilon_{x}^{s}-\frac{2\sqrt{2}}{\omega }\cos\left(\theta_{a}\right)\varepsilon_{x}^{s}}{T\varepsilon_{x}^{s}}\\ & =\frac{-\frac{\sqrt{2}}{3}\alpha t_{a}^{3}-\frac{2\sqrt{2}t_{c1}}{\omega }\cos\left(\theta_{a}\right)}{T} \end{array},$$

The above equation is further transformed into the following equation:

(21) $$Rate=\frac{-\frac{\sqrt{2}}{24}\theta_{a}\alpha \frac{t_{a}}{t_{c1}}-\frac{\sqrt{2}}{2\pi }\cos\left(\theta_{a}\right)}{2\frac{t_{a}}{t_{c1}}+\frac{t_{s}}{t_{c1}}+\frac{3}{4}},$$

According to Equation (21), there are two methods to reduce the residual drift ratio. The residual drift ratio can be reduced by extending the residence time at each position or changing the acceleration process of the turning mechanism. It is easier to extend the residence time at each position in practical applications, but it is more difficult to find the optimal acceleration process of the turning mechanism. The residual drift ratio is reduced by prolonging the residence time at each position.

Let $c_{1}=-\frac{\sqrt{2}}{24}\theta_{a}\alpha \frac{t_{a}}{t_{c1}}-\frac{\sqrt{2}}{2\pi }\cos\left(\theta_{a}\right),c_{2}=2\frac{t_{a}}{t_{c1}}+\frac{3}{4}$; then, Equation (21) can be simplified as follows:

(22) $$Rate=\frac{c_{1}}{\frac{t_{s}}{t_{c1}}+c_{2}},$$

When the angular acceleration and angular velocity of the turning mechanism are constant, *c*_1_ and *c*_2_ are constants. According to Equation (22), prolonging the residence time at each position can significantly reduce the residual drift ratio. Suppose that the residence time of the turning mechanism in positions *A* and *D* is *t_s_*_1_, and the residence time in positions *B* and *C* is *t_s_*_2_; then, in a modulation period, four times will the turning mechanism remain in four stay positions. The sum of the attitude error caused by the bias drift of the gyroscope is as follows:

(23) $$\sum \Psi_{pk}=\left[\begin{array}{c} \sqrt{2}\left(t_{s1}-t_{s2}\right)\varepsilon_{x}^{s}\\ \sqrt{2}\left(t_{s1}-t_{s2}\right)\varepsilon_{y}^{s}\\ 2\varepsilon_{z}^{s}\left(t_{s1}+t_{s2}\right) \end{array}\right],$$

Taking the east direction as an example, the attitude angle error caused by the zero drift *ε* of the east gyroscope is as follows:

(24) $$\int \varepsilon_{E}^{n}dt=\left(-\frac{\sqrt{2}}{3}\alpha t_{a}^{3}-\frac{2\sqrt{2}t_{c1}}{\omega }\cos\left(\theta_{a}\right)+\sqrt{2}\left(t_{s1}-t_{s2}\right)\right)\varepsilon_{x}^{s},$$

If $-\frac{\sqrt{2}}{3}\alpha t_{a}^{3}-\frac{2\sqrt{2}t_{c1}}{\omega }\cos\left(\theta_{a}\right)+\sqrt{2}\left(t_{s1}-t_{s2}\right)=0$, the following is true:

(25) $$\Delta t=t_{s1}-t_{s2}=\frac{1}{3}\alpha t_{a}^{3}+\frac{2}{\omega }\cos\left(\theta_{a}\right),$$

Then, the east attitude angle error is zero, and the residual drift ratio is also zero, reaching the optimal compensation state.

## 4. Simulation Analysis and Determination of Optimal Modulation Scheme

### 4.1. Simulation Analysis

It can be seen from the theoretical analysis that the gyro bias drift is the main factor that causes a decline in the positioning accuracy. Therefore, the best modulation scheme can be selected by simulating and analyzing the modulation effect of the above scheme on the gyro bias drift. Matlab numerical simulation software was selected to simulate and analyze the above scheme. The parameter setting is shown in Table 1 and the simulation time was 72 h.

**Figure 6:**
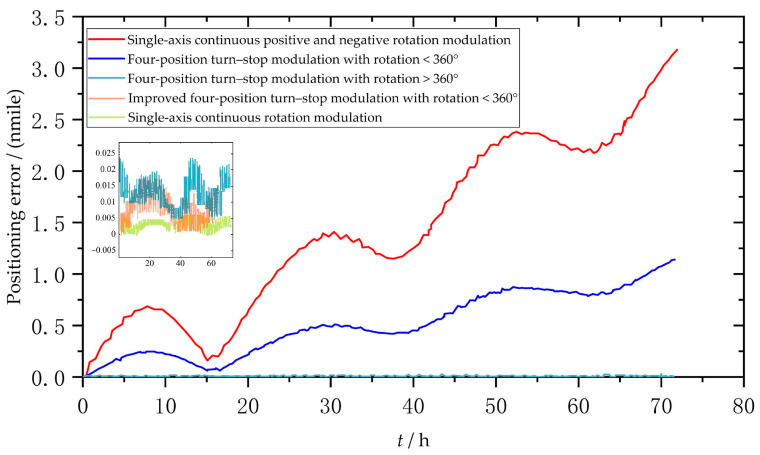
Positioning error caused by *ε* in different modulation schemes.

In each scheme, the positioning error caused by the constant drift error of the gyroscope in the direction of the vertical rotation axis is shown in Figure 6. In the simulation time of 72 h, the error of the single-axis continuous rotation modulation scheme was the minimum, only 0.005 nmile. The single-axis continuous positive and negative rotation scheme had the maximum error, exceeding 3 nmile. The error of the four-position turn–stop scheme with >360° was similar to that of the improved four-position turn–stop scheme with <360°, which was about 0.02 nmile. The four-position turn–stop scheme with <360° had an error of approximately 1.2 nmile.

### 4.2. Determination of Optimal Modulation Scheme

Based on the above theory and simulation analysis, it can be seen that the error of the single-axis continuous rotation modulation scheme is the minimum. However, under the condition of a long endurance operation, the bias drift error will diverge over time, which will seriously affect the system accuracy, so this scheme should not be adopted.

The single-axis continuous positive and negative rotation scheme has the worst accuracy, which is inconsistent with the theoretical analysis. The reason is that the gyro bias drift error cannot be completely suppressed due to the asymmetry of the rotation process in the whole cycle, and the gyro bias drift error remains due to the alternating residence time of the positive and negative rotations, which ultimately affects the accuracy of the navigation system, so this scheme should not be used.

The four-position turn–stop scheme >360° can completely modulate the gyro bias drift in the vertical axis direction within a complete modulation period, and this scheme will not produce other error-influencing factors that diverge over time. This scheme can be used as an alternative.

The inertial sensor drift error of the four-position turn–stop scheme with <360° can be well compensated, but the gyro drift error cannot be effectively compensated, which will eventually affect the accuracy of the navigation system, so this scheme should not be used.

The improved four-position turn–stop scheme with <360° cannot effectively compensate the gyro bias drift error, but after the theoretical adjustment of the four-position residence time of this scheme, the residual gyro bias drift error within the residence time just cancels out the residual gyro bias drift error in the process of uniform rotation, making the gyro bias drift error completely modulated in a complete modulation cycle. This scheme can also be used as an alternative.

In summary, the four-position turn–stop scheme with >360° and the improved four-position turn–stop scheme with <360° are alternatives. The compensation effect of both schemes is good, and the system accuracy is basically equivalent. However, in practical applications, the four-position turn–stop scheme with >360° requires the installation of conductive slip rings on the turning mechanism, which increases the cost of the navigation system. On the other hand, the conductive slip ring may introduce external errors when it works for a long voyage, thus affecting the system accuracy. The improved four-position turn–stop scheme with <360° does not require additional conductive slip rings, so the system cost is relatively low, and it is relatively easy to achieve. Therefore, the improved four-position turn–stop scheme with <360° is the best single-axis rotation modulation scheme in practical engineering applications.

## 5. Research on Error Modulation Experiment and Engineering Application

### 5.1. Experimental Study on the Improved Four-Position Turn–Stop with <360°

**Figure 7:**
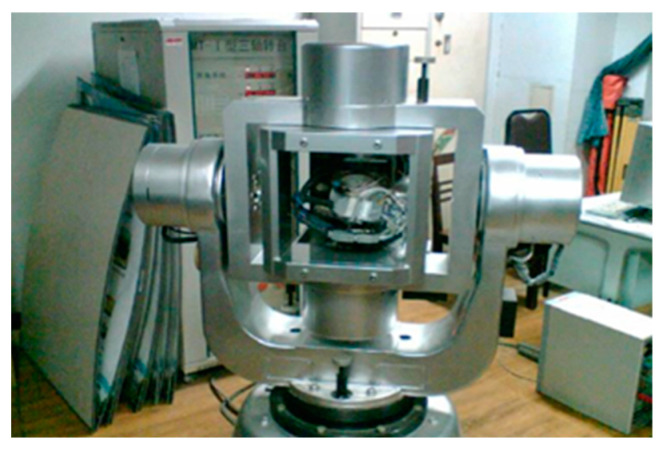
SMT-1 three-axis turntable.

The experiment selected the SMT-1 three-axis turntable as the working platform for testing the inertial sensor, as shown in Figure 7. During the experiment, the platform provided the reference motion of the gyroscope and recorded and stored the actual angle change curve. The output of the gyroscope in the inertial measurement component was input to the signal acquisition computer through the RS-232 serial port, and data processing and comparison were carried out in the later stage.

**Figure 8:**
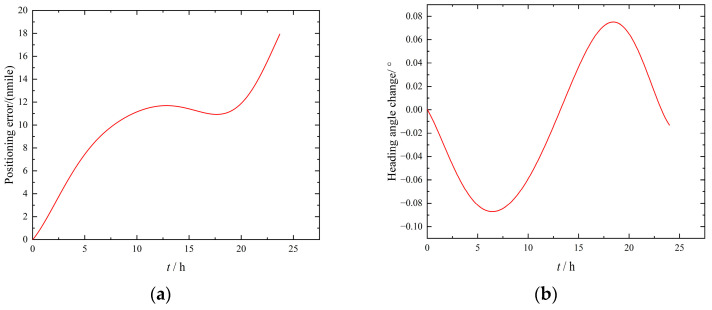
Static experiment results of IMU. (**a**) Positioning error. (**b**) Heading angle change.

In order to verify the error modulation effect of the improved four-position turn–stop scheme with <360°, the static performance index of the INS needed to be determined first. The error test results of the IMU 24 h static test under the condition that the turning mechanism does not rotate are shown in Figure 8. It can be seen from Figure 8a that when the IMU remained stationary, that is, when the system error was not modulated, the positioning error gradually accumulated within 12 h after the start of the experiment and slowly decreased between 12 and 17 h, but then the positioning error increased again, and the final positioning error was 18 nmile in 24 h. It can be seen from Figure 8b that the heading angle presented a similar sine wave change; it showed a trend of first increasing, then decreasing to zero, then increasing, then decreasing to zero, and then increasing within 24 h. The final heading angle changed to 0.02°, and the amplitude of oscillation was 0.08°.

**Figure 9:**
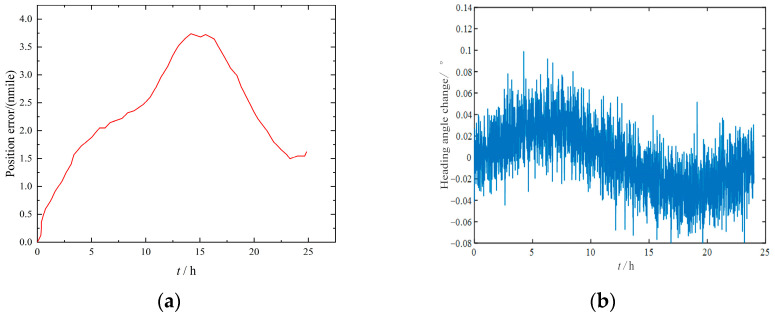
Experimental results of the improved four-position turn–stop scheme with <360°. (**a**) Positioning error. (**b**) Heading angle change.

The angular velocity and angular acceleration of the improved four-position turn–stop scheme with <360° were selected as shown in Table 1. The residence time at each stop position was 100 s, and the total test time was 24 h. This experimental scheme can completely modulate the influence of inertial sensor bias error on the final error of the navigation system. The final positioning error and heading angle change in this scheme are shown in Figure 9. It can be seen from Figure 9a that the positioning error gradually increased within 15 h after the start of the experiment, and then decreased gradually. The final 24 h positioning error was 3.7 nmile, which is more than four times higher than the static experimental positioning accuracy. It can be seen from Figure 9b that the change in heading angle within 24 h showed a trend of first increasing, then decreasing to zero, then increasing, and then decreasing. The final heading angle changed by 0.06°, which is 0.02° less than the static error. In addition, due to the superposition of the indexing mechanism and the inertial navigation itself, the experimental system had significant heading angle noise.

### 5.2. Analysis on the Compensation Effect of Shearer Operating Attitude Perception Error

**Figure 10:**
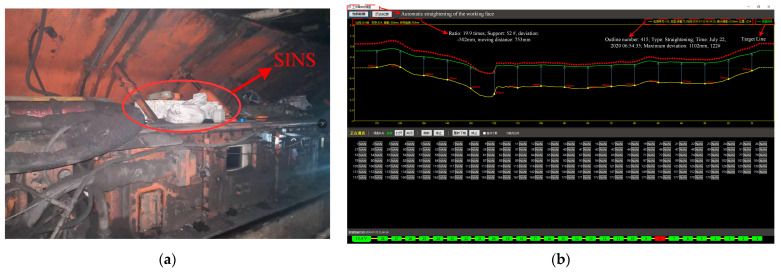
Site layout of SINS of shearer and shearer path diagram. (**a**) Field application of SINS for shearer. (**b**) Shearer path diagram.

The SINS of the coal shearer in the No.211 working face of a mine is mainly used to sense the operating posture of the coal shearer in real time and guide the automatic straightening of the scraper conveyor, as shown in Figure 10. It is regarded as a horizontal coal seam, since the average dip angle of the coal seam is less than 1°. The shape of the scraper conveyor depends on the output coordinates of the shearer SINS in *X* and *Y* axes. First, the compensation effect of the positioning error of the shearer SINS was investigated by analyzing the shape of the scraper conveyor.

**Figure 11:**
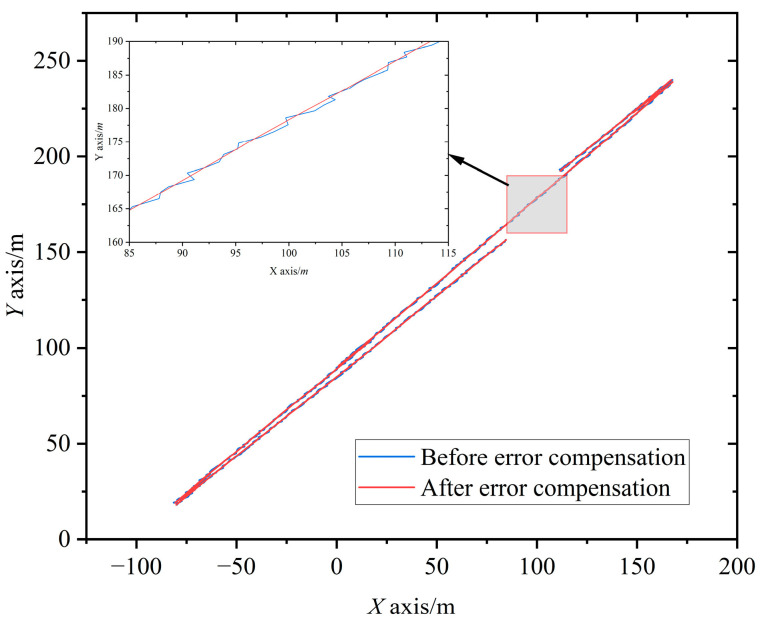
Running track of shearer.

The automatic straightening interface of the scraper conveyor is shown in Figure 10b. The yellow line in the figure is the reference line, the green line is the actual path, and the red line is the straightening target. The output positioning information of the SINS shows that most of the scraper conveyor deviated from the reference line by about 800 mm, which is consistent with the cutting depth of the shearer by 0.8 m. However, the scraper conveyor between No. 118–150 supports presented an obvious bending shape, with an average deviation of 130 mm from the cutting depth of the shearer. The deviation between the No. 122 support and the reference line was the largest, reaching 1102 mm, and the deviation from the cutting depth of the shearer was about 300 mm. Figure 11 shows the operation track of the shearer before and after error compensation. After the error compensation, the average deviation of the scraper conveyor between No. 118–150 supports was 822 mm from the reference line, and the average deviation of the shearer section depth was 22 mm. The straightening error of the scraper conveyor after the error compensation, that is, the plane positioning error, was about 17% of that before the compensation. The positioning accuracy of the shearer SINS was significantly improved.

**Figure 12:**
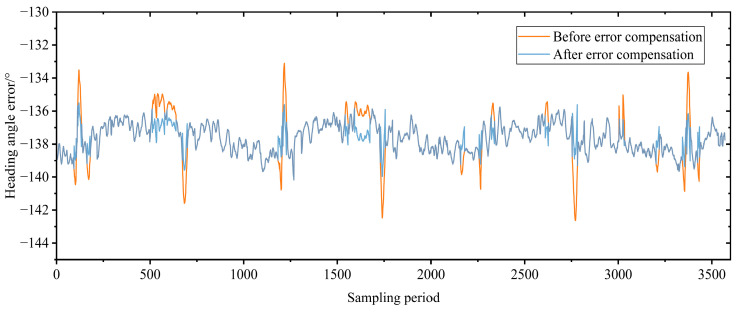
Shearer heading angle change.

Since the roll angle and pitch angle of the shearer fluctuate relatively little, the heading angle (which has a larger fluctuation) was selected to study the attitude angle error compensation effect of the SINS. After removing invalid data, the output fluctuation of 3571 heading angles during the period 00:00:01–07:16:26 on 22 July 2020 is shown in Figure 12. Before error compensation, the heading angle fluctuated significantly in this period, with a maximum fluctuation value of 5.03° and a final cumulative error of 0.92°. After error compensation, the characteristics of heading angle fluctuation were significantly reduced: the maximum fluctuation value was 2.56° and the final cumulative error was 0.69°. After compensation, the heading angle error was 75% of that before compensation, and the attitude angle perception accuracy of the shearer SINS was also significantly improved.

## 6. Conclusions

(1) This paper theoretically analyzed the propagation rules of inertial sensor drift error, scale factor error, and installation error in single-axis continuous rotation, single-axis continuous positive and negative rotation, >360° four-position stop, <360° four-position stop, and the <360° improved four-position stop error modulation scheme. It was proved theoretically that the <360° improved four-position stop scheme can eliminate the influence of gyro drift in the vertical rotation axis direction. Moreover, it achieves a relatively good error self-compensation effect.
(2) In this paper, five single-axis rotation error modulation schemes were studied through simulation analysis. The research shows that the positioning error of the improved four-position turn stop scheme <360° was about 0.01 nmile within 72 h of simulation time. After a comprehensive analysis, this scheme was determined to be the best rotation modulation scheme in practical engineering applications. Based on the optimal rotation modulation scheme, a single-axis rotation error modulation experiment was carried out, which verified that the scheme can effectively improve the sensing accuracy of SINS.
(3) The field application of shearer operation attitude perception was analyzed and studied. The field application showed that the plane positioning error after error compensation was about 17% of that before compensation, and the heading angle error was 75% of that before compensation, which verifies the effectiveness of the error compensation algorithm in this paper, and the shearer operation attitude perception accuracy has been significantly improved.
(4) Although the single-axis rotation error modulation of the SINS of the shearer was studied in this paper, there is a lack of specific research on the rotation error effect of the turning mechanism in the vibration environment, which will affect the system error self-compensation effect of the single-axis rotation modulation IMU applied to the long flight and the selection of the optimal attitude solution structure of the shearer. Therefore, the rotation error effect in the vibration environment needs to be further explored.

## Figures and Tables

**Table 1:** Model and turning mechanism parameter table.

Parameter	Value
$\varepsilon_{x}^{s}=\varepsilon_{y}^{s}=\varepsilon_{z}^{s}$	0.01°/h
*K_gx_* = *K_gy_* = *K_gz_*	10 ppm
*E_gxy_*	−10″
*E_gxz_*	−20″
*E_gyx_*	15″
*E_gyz_*	−10″
*E_gzx_*	20″
*E_gzy_*	5″
*ω*	6°/s
*α*	10°/s^2^

## Data Availability

All data and code used or analyzed in this study are available from the corresponding author on reasonable request.
